# Coenzyme Q10: A Key Antioxidant in the Management of Diabetes-Induced Cardiovascular Complications—An Overview of Mechanisms and Clinical Evidence

**DOI:** 10.1155/2024/2247748

**Published:** 2024-03-15

**Authors:** Fatemeh Samimi, Nasim Namiranian, Ali Sharifi-Rigi, Morvarid Siri, Omid Abazari, Sanaz Dastghaib

**Affiliations:** ^1^Diabetes Research Center, Shahid Sadoughi University of Medical Sciences, Yazd, Iran; ^2^Department of Biochemistry, School of Medicine, Shiraz University of Medical Sciences, Shiraz, Iran; ^3^Autophagy Research Center, Department of Clinical Biochemistry, School of Medicine, Shiraz University of Medical Sciences, Shiraz, Iran; ^4^Department of Clinical Biochemistry, School of Medicine, Shahid Sadoughi University of Medical Sciences and Health Services, Yazd, Iran; ^5^Endocrinology and Metabolism Research Center, Shiraz University of Medical Sciences, Shiraz, Iran; ^6^Autophagy Research Center, Shiraz University of Medical Sciences, Shiraz, Iran

## Abstract

**Background:**

Diabetes mellitus (DM) presents a significant global health challenge with considerable cardiovascular implications. Coenzyme Q10 (CoQ10) has gained recognition for its potential as a natural antioxidant supplement in the management of diabetes and its associated cardiovascular complications.

**Aim:**

This comprehensive review systematically examines the scientific rationale underlying the therapeutic properties of CoQ10 in mitigating the impact of diabetes and its cardiovascular consequences. The analysis encompasses preclinical trials (*in vitro* and *in vivo*) and clinical studies evaluating the efficacy and mechanisms of action of CoQ10. *Result & Discussion*. Findings reveal that CoQ10, through its potent antioxidant and anti-inflammatory attributes, demonstrates significant potential in reducing oxidative stress, ameliorating lipid profiles, and regulating blood pressure, which are crucial aspects in managing diabetes-induced cardiovascular complications. CoQ10, chemically represented as C_59_H_90_O_4_, was administered in capsule form for human studies at doses of 50, 100, 150, 200, and 300 mg per day and at concentrations of 10 and 20 *μ*M in sterile powder for experimental investigations and 10 mg/kg in powder for mouse studies, according to the published research. Clinical trials corroborate these preclinical findings, demonstrating improved glycemic control, lipid profiles, and blood pressure in patients supplemented with CoQ10.

**Conclusion:**

In conclusion, CoQ10 emerges as a promising natural therapeutic intervention for the comprehensive management of diabetes and its associated cardiovascular complications. Its multifaceted impacts on the Nrf2/Keap1/ARE pathway, oxidative stress, and metabolic regulation highlight its potential as an adjunct in the treatment of diabetes and related cardiovascular disorders. However, further extensive clinical investigations are necessary to fully establish its therapeutic potential and assess potential synergistic effects with other compounds.

## 1. Introduction

### 1.1. Diabetes Mellitus (DM)

DM is a significant metabolic disorder characterized by elevated blood sugar levels, resulting from a complex interplay of genetic and environmental factors. It has emerged as a major chronic noncommunicable disease that profoundly impacts the health and well-being of individuals worldwide [1]. According to the International Diabetes Federation (IDF), the number of people living with diabetes was estimated at 536.6 million in 2021, and this figure is projected to reach 783.2 million by 2045 [2]. Consequently, diabetes has become an increasingly pressing concern both in clinical practice and public health initiatives [3]. Diabetes is associated with various complications, including heart disease, nephropathy, neuropathy, retinopathy, cataracts, and a range of other health issues [4]. Among these complications, cardiovascular disease stands out as one of the most prevalent and severe consequences of diabetes, being a leading cause of illness and mortality in diabetic individuals [5]. The primary goal of diabetes treatment is to manage and lower blood glucose levels in affected patients, which is typically achieved through the use of antidiabetic medications and lifestyle adjustments such as weight management, healthy dietary choices, and increased physical activity [6]. Presently, the mainstay of diabetes treatment involves insulin and hypoglycemic medications, which often entail side effects such as hypoglycemic episodes, headaches, dizziness, nausea, and hypersensitivity reactions. Furthermore, these pharmaceutical interventions often fall short in providing long-term relief from diabetes symptoms [5, 7]. Hence, there is a growing need to identify effective, natural compounds with fewer adverse effects for the management of diabetes and its associated complications [8]. In light of the rising prevalence of the diabetes epidemic, its social and economic ramifications, and the limitations of conventional pharmaceuticals, increasing attention has been directed toward the exploration of antioxidant supplements derived from natural sources, which hold promise for the treatment of diabetes and its complications [9]. The present study aimed to examine the antioxidant properties of COQ10 and its potential beneficial effects on oxidative stress induced by diabetes and associated cardiovascular complications.

## 2. Coenzyme Q10 (COQ10)

COQ10 is a notable supplement utilized in the management of various health conditions, including cardiovascular diseases, diabetes, neurological disorders, and metabolic irregularities such as hyperlipidemia and hypertension, as well as mitochondrial diseases [10]. Extensive research has highlighted the beneficial impacts of CoQ10 in safeguarding against diabetes and its associated cardiovascular complications. These effects are purportedly linked to its antioxidant properties, anti-inflammatory attributes, antihypertension, hypoglycemic effects, and the ability to counteract hyperlipidemia aimed at ameliorating diabetes and its cardiovascular ramifications [11–13].

This review endeavors to elucidate the scientific basis supporting the therapeutic properties of CoQ10 as a promising antioxidant in the context of managing diabetes and its consequential cardiovascular complications. Research has primarily ascribed the advantageous impacts of CoQ10 to its well-documented bioenergetic and antioxidant properties [14]. Although the vast majority of individuals tolerate coenzyme Q10 without any side effects, it is possible to experience mild adverse effects such as nausea, vomiting, diarrhea, stomach upset, and appetite loss. It may elicit allergic skin rashes in certain individuals. In addition, it may cause a drop in blood pressure [15]. Furthermore, it seeks to provide an overview of the specific effects and underlying molecular mechanisms through which CoQ10 may contribute to the improvement of diabetes and its associated cardiovascular issues.

### 2.1. CoQ10's Beneficial Impacts on Diabetes and Associated Cardiovascular Consequences

**Figure 1:**
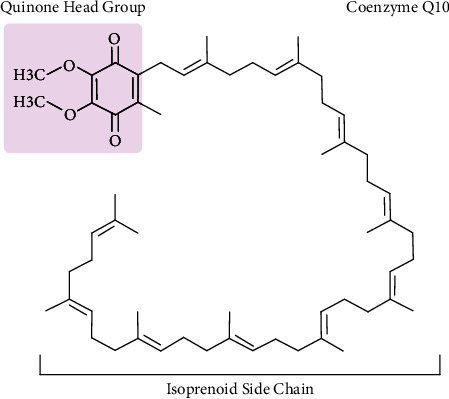
The chemical structure of coenzyme Q10 comprises a benzoquinone ring bonded to an isoprenoid side chain that consists of ten connected isoprenyl groups.

2,3-Dimethoxy-5-methyl-6-polyprenyl-1,4-benzoquinone, or CoQ10, is a lipophilic substance that resembles a vitamin. It is found in many different types of life and functions as a strong agent with anti-inflammatory, antiapoptotic, and antioxidant properties [16]. CoQ10 is made up of two key elements, including a benzoquinone ring that aids in the transmission of electrons to the mitochondrial membrane's respiratory chain. This aids adenosine triphosphate production, which helps cells generate energy. The other component is an isoprenoid chain, which provides the molecule's hydrophobic characteristics (Figure 1). Isoprenyl chain lengths vary between species. For example, humans contain CoQ10, which has 10 isoprenyl units, but rats normally have CoQ9 [17]. In human cells, the quinone head of CoQ is joined to a chain of nine (CoQ9) or ten isoprene units (CoQ10) to form CoQ. Although coenzyme Q10 makes up a sizable component of total CoQ and can rise in response to oral treatment, coenzyme Q9 is more prevalent in mice and rat tissues than it is in human tissues. It was discovered that CoQ10 is absorbed into the brain. Moreover, the exogenous lipid significantly increases the amount of CoQ9 that is produced internally [18]. CoQ10 is essential for protecting cells from oxidative damage and increasing cellular energy. Furthermore, CoQ10 may be used as an adjuvant therapy in the management of infectious disorders, according to certain research. It is commonly known that oxidative stress and inflammation play important roles in bacterial and viral illnesses. CoQ10 works to prevent viral infections by acting as an antioxidant and reduces inflammation, either directly or indirectly. In order to fight a disease, immune cells release reactive oxygen species (ROS) and cytokines during an infectious process. The direct antioxidant effect of CoQ10 can prevent lipoperoxidation, which is a product of these ROS. By suppressing the expression of the nuclear factor kappa B (NF-kB) gene, CoQ10 may have anti-inflammatory properties by lowering the synthesis of interleukin 1 (IL-1), IL-6, and tumor necrosis factor *α* (TNF-*α*) gene expression [19]. It is primarily prevalent in the heart, liver, kidneys, and pancreas and is present in cell membranes throughout the body, particularly in the inner mitochondrial membrane [19, 20]. Despite the fact that the human body naturally produces CoQ10, the synthesis of this vitamin-like substance decreases with age, which may result in deficits in elderly people [21]. Coenzyme Q10 (ubiquinol) is present in the cellular membrane and functions to prevent oxidation of lipoproteins and the lipid membrane by being enzymatically maintained in its reduced state. However, as people age, their body produces less coenzyme Q10 [22, 23]. The human lung, heart, spleen, liver, and kidney have the maximum concentration of CoQ10 around age 20, after which it progressively declines with more aging. CoQ10 is present in small amounts in a variety of food sources and is also produced industrially in labs for use as a dietary supplement or medicine [24].

While diabetes can lead to a range of health issues, vascular complications are primarily responsible for the majority of morbidity and mortality rates in diabetic patients [25]. Research indicates that diabetes contributes to vascular senescence, which is linked to persistent inflammation and heightened oxidative stress resulting from elevated blood sugar levels. These factors play a pivotal role in the development of endothelial dysfunction [26]. Type 2 diabetes, the prevailing form of diabetes, is associated with specific manifestations including dyslipidemia, atherosclerosis, and hypertension [27]. In the context of T2D, insulin resistance and obesity collectively contribute to increased fasting triglyceride (TG) and LDL cholesterol levels, while simultaneously causing a decline in HDL cholesterol concentrations. The accumulation of lipids exacerbates the progression of diabetes, potentially leading to complications such as heart failure, myocardial infarction, stroke, or cardiovascular death [5, 12].

In cardiovascular diseases (CVDs), the prolonged use of therapies such as statins, a prevalent class of lipid-lowering medications, can effectively manage dyslipidemic complications but may concurrently lower serum levels of CoQ10 over time. CoQ10 is an essential component of mitochondrial respiration and energy production within muscle cells. Statins work by inhibiting the enzyme HMG-CoA reductase, which is a crucial enzyme regulating the synthesis of cholesterol as well as CoQ10. It appears that gemfibrozil (Lopid) as a lipid-lowering drug may decrease coenzyme Q10 levels and propranolol, and metoprolol may inhibit coenzyme Q10-dependent enzymes. This inhibition leads to a decrease in CoQ10 levels in both muscle tissue and serum [28]. CoQ10 assumes a crucial role in addressing diabetes and its associated cardiovascular complications through a variety of mechanisms. Its advantageous effects in managing these conditions stem from its potent antioxidant and anti-inflammatory properties, its capacity to counteract abnormal lipid levels, and its essential role in maintaining mitochondrial function [29]. In the following sections, we will explore a diverse range of recent examinations, including *in vivo*, *in vitro*, and clinical trials, to elucidate the underlying mechanisms through which CoQ10 offers protection against diabetes-related cardiovascular complications. These findings are summarized in Tables 1 and 2.

### 2.2. The Role of CoQ10 in Mitigating Oxidative Stress and Its Impact on Diabetes-Induced Cardiovascular Complications

The heightened prevalence of diabetes and the consequent increase in mortality rates prompted the World Health Organization to issue a grave warning, particularly emphasizing its impact on developing nations [59]. Diabetes is frequently accompanied with increased oxidative stress, which contributes significantly to the onset and progression of cardiovascular diseases. CoQ10, a powerful antioxidant, has been studied for its ability to prevent oxidative damage in diabetes patients [60]. According to research, CoQ10 supplementation may help decrease oxidative stress by neutralizing reactive oxygen species and minimizing oxidative damage to cells and tissues. CoQ10's antioxidant activity has been linked to protection against a variety of diabetes-related cardiovascular complications, including endothelial dysfunction, atherosclerosis, and myocardial damage [61]. Furthermore, CoQ10 supplementation has been proven to increase endothelial function, reduce inflammation, and boost mitochondrial activity, all of which contribute to its potential cardioprotective benefits in diabetic complications [62]. Overall, previous research has shown that CoQ10 is important in countering oxidative stress and has a positive influence on reducing diabetes-induced cardiovascular complications. In this section, we will examine how CoQ10 influences cardiovascular complications induced by diabetes by regulating oxidative stress.

Dyslipidemia, which is defined by elevated levels of triglycerides (TG), total cholesterol (TC), low-density lipoprotein cholesterol (LDL-C), and low levels of high-density lipoprotein cholesterol (HDL-C), significantly increases the risk of atherosclerosis and cardiovascular disease in diabetic individuals. Atherosclerosis pathogenesis includes mechanisms such as hyperglycemia, nonenzymatic glycosylation of lipids, activation of signaling pathways leading to the generation of proinflammatory cytokines/chemokines, and enhanced oxidative stress [63]. Oxidative stress, defined as an imbalance between the formation of reactive oxygen species (ROS) and the body's innate antioxidant defense systems, causes protein and lipid peroxidation, as well as DNA mutagenesis [12, 64, 65]. ROS plays a key role in inflammatory responses, vascular tone alterations, and LDL-C oxidation. Diabetes and its consequences, such as cardiovascular disease and dyslipidemia, cause an increase in ROS generation inside the arterial wall [66]. NADPH oxidase, xanthine oxidase, mitochondrial enzymes, and uncoupled endothelial nitric oxide synthase (eNOS) are all important ROS generators in blood vessels [63]. NADPH oxidases (NOX) are considered the principal ROS producers in the cardiovascular system, contributing to the formation of superoxide anions by transferring electrons from NADPH to molecular oxygen. NOX activity in macrophages enhances the development of oxLDL (oxidized low-density lipoprotein), which then boosts ROS production [67]. Xanthine oxidase, which is released from the liver into the circulation, binds to the endothelial cell membrane, causing the production of superoxide anions and hydrogen peroxide via the use of molecular oxygen. Its levels are shown to be higher in human atherosclerotic plaque [68]. Furthermore, mitochondrial enzymes normally create superoxide anions under healthy situations, whereas mitochondrial malfunction or the failure of antioxidant systems can result in pathological states defined by excessive ROS generation [69].

Several studies have shown that CoQ10 can effectively reduce hyperglycemia, total cholesterol (TC), LDL-C, and triglyceride levels in diabetic rats while increasing HDL-C concentrations [30, 40, 41]. Furthermore, in a study of hypertensive rats, a treatment with 10 mg/kg of CoQ10 over 6 weeks significantly lowered blood pressure, TC, LDL-C, tumor necrosis factor *α* (TNF-*α*), and the lipid peroxidation product malondialdehyde (MDA). It also raised the levels of total antioxidant capacity (TAC) [34]. Tsai et al. demonstrated that CoQ10 significantly mitigated the oxidative stress induced by oxLDL, thus elevating the activity of eNOS, catalase (CAT), and superoxide dismutase (SOD) enzymes. This finding suggests its potential to protect against atherogenesis through NO-related pathways in human umbilical vein endothelial cells [22].

Khosrowbeygi et al. found that consuming 100 mg/day of CoQ10 raised TAC, catalase activity, and QUICKI, while decreasing oxidative stress and fasting blood sugar (FBS) levels in women with T2DM [54]. Similarly, Hosseinzadeh-Attar et al. reported that giving 200 mg/day of CoQ10 to T2DM patients resulted in a substantial decrease in HbA1c levels, indicating better glycemic management. Furthermore, a significant reduction in serum LDL-C levels was detected, which may provide protection against atherosclerotic plaques and consequent cardiovascular problems in T2DM patients [50]. Kadhim Mohammed-Jawad et al. discovered that a 150 mg/day of CoQ10 supplementation over 8 weeks resulted in a noticeable drop in blood cholesterol and LDL-C levels, with a substantial influence on HDL-C in 38 individuals with T2DM [47]. These findings support the findings of Kolahdouz Mohammadi et al., who provided 200 mg/day of CoQ10 to T2DM patients for 12 weeks [70]. Emerging data show that oxidative stress contributes to hyperglycemia, insulin resistance, and beta cell dysfunction [71]. CoQ10 insufficiency is linked to beta cell dysfunction, as well as reduced glucose and fatty acid metabolism in the liver, resulting in diminished insulin activity. CoQ10 supplementation lowers blood glucose levels and increases insulin sensitivity by modulating the insulin signaling pathway, which includes the phosphorylation of insulin receptor substrate (IRS) proteins and glucose transporter 2 (GLUT2), as well as improving the lipid profile and adipocytokines [72, 73]. CoQ10 promotes fatty acid oxidation by activating AMPK and PPAR*α*. As a result, this mechanism boosts lipoprotein lipase and apolipoprotein A-V (APO-AV) gene expression, potentially lowering TG and VLDL levels [74, 75]. PPAR*α* inhibits sterol regulatory element-binding protein (SREBP) maturation, thus reducing fatty acid and triglyceride production and increasing the size of LDL-C particles. This size growth is especially beneficial in preventing vascular problems in diabetic patients [75]. Activating PPAR*α* can decrease cellular inflammation and oxidative stress by inhibiting the AP-1 and NF-*κ*B signaling pathways [76]. Hodgson et al. found that daily treatment with 200 mg/day of CoQ10 over a 12-week period in T2DM patients resulted in a substantial drop in both systolic and diastolic blood pressure, as well as a reduction in HbA1c levels [43]. CoQ10 supplementation has demonstrated excellent antihypertensive effects in coronary artery disease, leading researchers to conclude that CoQ10 intake can effectively decrease blood pressure in hypertensive individuals [77]. In clinical hypertension, CoQ10 appears to increase coronary vasodilation and endothelial function [72].

Nitric oxide (NO), a critical vasoprotective element in the endothelium produced by eNOS, may become dysfunctional under conditions of oxidative stress, potentially leading to the inactivation of NO by an excess of superoxide anions. CoQ10 may effectively reduce blood pressure by decreasing peripheral resistance through the augmentation of NO availability [78].

**Figure 2:**
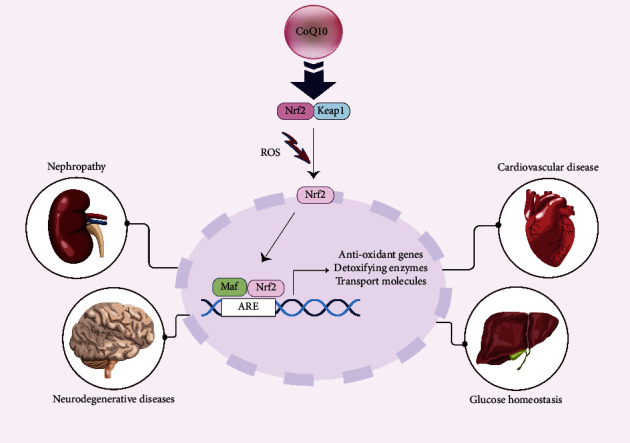
The Nrf2/Keap1/ARE pathway has a role in a wide range of tissues.

In addition, Lee et al. found that supplementation with 300 mg/day of CoQ10 significantly improved the activities of antioxidant enzymes and reduced inflammation in patients with coronary artery disease (CAD) during statin therapy [46]. Yen et al. demonstrated that 100 mg/day of CoQ10 supplementation led to a significant increase in catalase (CAT) and glutathione peroxidase (GPx) activity in patients with T2DM [57]. Moreover, a systematic review and meta-analysis revealed that CoQ10 supplementation significantly decreased malondialdehyde (MDA), while also increasing total antioxidant capacity (TAC) and superoxide dismutase (SOD) activity [79]. The primary antioxidant systems in the endothelial-vascular system involve essential antioxidant enzymes such as SOD, CAT, GPx, and the nitric oxide molecule [66]. SOD functions by converting superoxide radicals into hydrogen peroxide, which are subsequently broken down by CAT and GPx. Moreover, GPx facilitates the reduction of various peroxides, including lipid peroxides and oxidized phospholipids [63]. These antioxidant enzymes serve as the initial defense against ROS, and a decline in their activities can lead to lipid peroxidation and oxidative damage to cells, particularly in individuals affected by diabetes and coronary artery disease [60, 80]. Oxidative stress has been recognized as a factor that plays a significant role in the onset of diabetes and its associated complications [81]. The nuclear factor erythroid 2-related factor 2 (Nrf2), a critical transcription factor, plays a pivotal role in combatting oxidative stress. In normal conditions, Nrf2 is sequestered in the cytoplasm through its binding to the repressor protein Keap1 (Kelch-like ECH-associated protein 1), resulting in the ubiquitination and degradation of Nrf2. Keap1 contains several cysteine residues that function as sensors of oxidative stress and act as negative regulators of Nrf2 [82]. Modification of cysteine residues in Keap1 leads to the translocation of Nrf2 into the nucleus, where it forms a heterodimer with a small Maf protein and binds to the antioxidant response element (ARE), ultimately inducing the expression of heme oxygenase 1 (HO-1) and other ARE-regulated genes such as CAT, SOD, and GPx [83]. In a study by Susana Siewert et al., a robust correlation between Nrf2 and HO-1 was demonstrated, with mRNA levels being significantly lower in the diabetic group than in the healthy control group. The findings of this study suggest a reduction in genes associated with antioxidant defense mechanisms in individuals with diabetes [84]. Given the close association between T2DM and oxidative damage, the involvement of the Nrf2/Keap1/ARE pathway in addressing this unresolved clinical issue has become a topic of significant interest. It is now evident that dysfunction of this pivotal antioxidant pathway plays a central role in the pathogenesis of diabetes and its diverse complications [85–87] (Figure 2).

Research findings have revealed the potential of CoQ10 in enhancing various physiological aspects in the context of diabetes. Specifically, in diabetic rat models, CoQ10 treatment led to a notable increase in Nrf2 expression and catalase activity within the liver tissue, accompanied by a significant reduction in MDA levels and considerable improvements in the lipid profile. These results indicate that the antioxidative effects of CoQ10 in diabetes might be attributed to the activation of the Nrf2/ARE pathway [88]. Moreover, Sun et al.'s study highlighted CoQ10's role in activating the Nrf2/Keap1/ARE pathway, consequently improving mitophagy and reducing damage to the kidneys in diabetic nephropathy [89]. Another study demonstrated that CoQ10 prevents diabetic nephropathy via prohibited mitochondrial dysfunction, proteinuria, and glomerular hyperfiltration [90]. In addition, Li et al. observed that CoQ10 facilitates the nuclear localization of Nrf2, enhancing cellular antioxidant defense and mitigating H_2_O_2_-induced neurotoxicity in pheochromocytoma (PC12) cells [91]. Further research suggested that CoQ10 supplementation significantly elevated Nrf2 and NQO1 expression in the hearts of elderly diabetic rats, thereby enhancing the heart function in elderly individuals with diabetes through the augmentation of endogenous antioxidant enzymes [92].

The reduction in Coenzyme Q10 concentration and antioxidant capacity observed in diabetes and its related cardiovascular complications underscores the significance of supplementing with potent antioxidants. Numerous previous studies have demonstrated that various antioxidants, including CoQ10, quercetin, curcumin, resveratrol, vitamin C, and vitamin E, offer preventive and therapeutic benefits in the context of diabetes and its associated cardiovascular complications [93]. Various studies have demonstrated a significant reduction in cyclic adenosine monophosphate (cAMP) and adenosine triphosphate (ATP) levels under conditions of oxidative stress [94, 95].

Recent research suggests that CoQ10 supplementation leads to an increase in cAMP production. cAMP serves as a crucial intracellular second messenger that activates AMPK, an energy sensor that, in turn, enhances the expression of sirtuin1 (Sirt1) [96]. Subsequently, Sirt1 augments the expression of Nrf2 by promoting its nuclear translocation and facilitating its binding to the ARE region. Nrf2, in turn, enhances the activity of mitochondrial electron transport chain complexes and upregulates the activity of antioxidant enzymes [88]. In this manner, CoQ10 serves to increase antioxidant activities through the induction of the Nrf2/Keap1/ARE pathway, thereby potentially alleviating the impact of diabetes and its associated cardiovascular complications [88, 92, 97, 98].

## 3. Conclusion and Future Perspectives

CoQ10 is recognized as a powerful antioxidant that demonstrates protective effects against a spectrum of diseases, including diabetes and associated cardiovascular complications. Current research suggests that CoQ10 operates through mechanisms that include the reduction of oxidative stress, anti-inflammatory actions, and the regulation of glucose and lipid metabolism. By modulating the Nrf2/Keap1/ARE pathway, CoQ10 can ameliorate diabetes-induced oxidative stress and subsequently stimulate the production of antioxidant enzymes. CoQ10 is generally considered to be pharmacologically safe, exhibiting good efficacy with minimal side effects. The limitations of CoQ10 supplementation in clinical trials include issues with absorption, bioavailability, and the heterogeneity of study designs, which make it challenging to identify its real impact. CoQ's limited bioavailability continues to be a significant obstacle to its application, despite extensive research into novel formulation strategies and the direct application of ubiquinol and ubiquinone, which is more accessible to cellular compartments and does not require cellular reduction. Despite these limitations, CoQ10 supplementation has shown promising results in certain clinical trials, which demonstrated a reduction in the relative risk of cardiac-related deaths in heart failure patients. However, it is important to acknowledge the conflicting results and the need for further research to elucidate the specific effects of CoQ10 and its potential value in clinical applications, especially in the context of oxidative stress. Future research should focus on addressing the limitations of CoQ10 supplementation, such as improving its bioavailability and conducting well-designed, homogenous clinical trials to determine its efficacy in various populations and disease conditions. However, additional studies are required to verify the effectiveness of CoQ10 and to elucidate the diverse mechanisms by which it can serve as a promising alternative in the prevention and treatment of diabetes and its associated cardiovascular complications. The disadvantage is that although studies on the effect of CoQ10 on the Nrf2/Keap1/ARE signaling pathway have been reported in animal experiments, the effect of this compound on the expression of genes involved in this pathway has not been reported in clinical trials. Therefore, large-scale clinical trials in this regard will contribute to a more comprehensive understanding of the full therapeutic potential of CoQ10. In addition, considering the ability of CoQ10 in different cellular signaling pathways to generate a stable cell condition, further research studies on the potential synergistic effects of CoQ10 with other natural or synthetic compounds and the study of its possible mechanisms can be one of the most interesting topics for future studies.

## Figures and Tables

**Table 1:** Preclinical trials of CoQ10 in the treatment of diabetes and its cardiovascular complications.

Study	Model	Treatment (powder)	Duration	Outcomes	Reference
*In vivo*	STZ-induced diabetic rats	10 mg/kg/day CoQ10	1 weeks	↓FBG, atherogenic index	[30]
				↓TG, TC, LDL, VLDL	
				↑HDL	
				↓BP	

*In vivo*	STZ-induced diabetic rats	10 mg/kg/day CoQ10	12 weeks	Improved LV performance and myocardial relaxation	[31]

*In vivo*	Alloxan-induced type 1 diabetic rats	15 mg/kg/day CoQ10	8 weeks	↓FBG, atherogenic index, cardiac risk ratio	[32]
				↓TG, TC, LDL, VLDL	
				↑HDL	

*In vivo*	Mouse model of type 1 diabetes	50 and 150 mg/kg/day	24 days	↓FBG, body weight	[33]
		CoQ10	3 days	↓Lipid peroxidation	

*In vivo*	l-NAME-induced hypertensive rats	10 mg/kg/day CoQ10	6 weeks	↓SBP, DBP, MAP	[34]
				↓TC, LDL, MDA, TNF-*α*	
				↑TAC	

*In vivo*	Type 2 diabetes db/db mouse	10 mg/kg/day CoQ10	10 weeks	↓ Superoxide generation	[35]
				↓Cardiac hypertrophy and fibrosis	
				↓Diastolic dysfunction	

*In vivo*	Mice diabetic cardiomyopathy	10 mg/kg, 3 times/week CoQ10	8 weeks	↓MDA	[36]
				↓Cardiac hypertrophy and fibrosis	
				↓Diastolic dysfunction	
				↓Expression of ANP	

*In vivo*	Hypertensive rats with fructose-fed	10 mg/kg/day CoQ10	4 weeks	↓BP	[37]
				↓ROS	
				↑SOD	

*In vivo*	STZ-induced diabetic rats	10 mg/kg/day CoQ10	2 weeks	↑CAT, GPx, GR	[38]

*In vivo*	Rats with myocardial infarction	Pretreated with 20 mg/kg/day CoQ10	1 weeks	↓LV infarct area	[39]
				↓TNF-*α*, IL-6	
				↑SOD, GPx	

*In vivo*	STZ-induced diabetic rats	10 mg/kg/day CoQ10	1 weeks	↑Erythrocyte GSH, GSH-Px activity	[40]
				↑HDL	
				↓Heart tissue lipid peroxidation, FBG, TC, TG	

*In vivo*	STZ-induced diabetic rats	10 mg/kg/day CoQ10	8 weeks	↓FBS, HbA1c, TC, TG, LDL	[41]
				↓Oxidation protein products	
				↓DNA impairment	
				↑NO, HDL	

*In vitro*	High glucose-induced EPC	10 *μ*M CoQ10	24 hours	↓ROS	[42]
				↓Caspase-3, ↑Bcl-2	
				↑Mitochondrial membrane potential	
				↑eNOS and HO-1 expression	

*In vitro*	oxLDL-induced oxidative stress in HUVEC	20 *μ*M CoQ10	24 hours	↓ROS	[22]
				↓IL-8, NF-*κ*B, COX-2 expression	
				↑CAT, SOD	
				↑eNOS, ↓iNOS	
				↑Bcl-2/BAX	

*In vitro*	AngII-induced oxidative stress in HUVEC	10 *μ*M CoQ10	24 hours	↓ROS	[23]
				↑NO, SOD, GPx	
				↓NOX2, ICAM-1, VCAM-1	

ANP, atrial natriuretic peptide; AngII, angiotensin II; BP, blood pressure; COX-2, cyclooxygenase-2; CAT, catalase; eNOS, endothelial nitric oxide synthase; EPC, endothelial progenitor cell; FBG, fasting blood glucose; GPx, glutathione peroxidase; GR, glutathione reductase; GSH, glutathione; HDL, high-density lipoprotein; HUVECs, human umbilical vein endothelial cells; IL-8, interleukin 8; ICAM-1, intercellular adhesion molecule 1; iNOS, inducible nitric oxide synthase; l-NAME, N-nitro-L-arginine methyl ester; LV, left ventricular; LDL, low-density lipoprotein; MDA, malondialdehyde; NF-*κ*B, nuclear factor kappa B; NOX2, NADPH oxidase 2; NO, nitric oxide; ROS, reactive oxygen species; SOD, superoxide dismutase; TNF-*α*, tumor necrosis factor *α*; TAC, total antioxidant capacity; TG, triglyceride; TC, total cholesterol; VCAM-1, vascular cell adhesion molecule 1; VLDL, very low-density lipoprotein.

**Table 2:** Clinical trial of CoQ10 in the treatment of diabetes and its cardiovascular complications.

Study group	Number of patients	Intervention (capsule)	Duration	Outcomes	Reference
Subjects with T2DM	37	200 mg/day CoQ10	12 weeks	↓HbA1c	[43]
				↓SBP, DBP	

Subjects with T2DM	56	200 mg/day CoQ10	12 weeks	↓HbA1c	[44]
				↓Weight, BMI	

Subjects with T2DM with LVDD	36	200 mg/day CoQ10	25 weeks	↓BP	[45]
				↓TG	

Subjects with CAD	51	300 mg/day CoQ10	12 weeks	↑CAT, SOD, GPx	[46]
				↓TNF-*α*, IL-6	

Subjects with T2DM	38	150 mg/day CoQ10	8 weeks	↓FBG, HbA1c	[47]
				↓TC, LDL, LPa	

Subjects with hyperlipidemia and MI	52	200 mg/day CoQ10	12 weeks	↑HDL	[48]
				↓TC, LDL	
				↓LDL/HDL, TC/HDL	
				↓SBP, DBP	

Subjects with diabetic neuropathy	62	200 mg/day CoQ10	12 weeks	↓HOMA-IR, CRP	[49]
				↑Insulin sensitivity, TAC	

Subjects with T2DM	64	200 mg/day CoQ10	12 weeks	↓HbA1c	[50]
				↓LDL	
				↓ADMA, nitrite and nitrate	

Subjects with T2DM	52	100 mg/day CoQ10	8 weeks	↓MDA levels	[51]

Obese and T2DM subjects with CHD	60	100 mg/day CoQ10	8 weeks	↓HOMO-IR, MDA	[52]
				↑TAC, GSH	

T2DM subjects with CHD	60	100 mg/day CoQ10	8 weeks	↓IL-6 and protein carbonyl levels	[53]

Subjects with T2DM	68	100 mg/day CoQ10	12 weeks	↓FBG	[54]
				↑CoQ10, CAT activity, TAC, and QUICKI	

Subjects with diabetic nephropathy	50	100 mg/day CoQ10	12 weeks	↓MDA and AGEs levels	[55]
				↑QUICKI	

Prediabetes subjects	80	200 mg/day CoQ10	8 weeks	↓HOMA-IR and oxygen-free radical	[56]

Subjects with T2DM	50	100 mg/day CoQ10	12 weeks	↓HbA1c, MES	[57]
				↑HDL	
				↑CAT, GPx	

Subjects with T2DM	27	100 mg/day CoQ10	12 weeks	↓HbA1c	[58]
				↓oxLDL	

AGEs, advanced glycation end products; ADMA, asymmetric dimethylarginine; BMI, body mass index; CRP, C-reactive protein; CHD, coronary heart disease; CAD, coronary artery disease; FMD, flow-mediated dilation; GSH, glutathione; HOMO-IR, homeostatic model assessment of insulin resistance; IL-6, interleukin 6; LPO, lipid peroxidation; LPa, lipoprotein(a); MES, medication effect score; QUICKI, quantitative insulin sensitivity check index.

## Abbreviations

AMPK: AMP-activated protein kinase
AP-1: Activator protein-1
ARE: Antioxidant response element
ATP: Adenosine triphosphate
CAD: Coronary artery disease
cAMP: Cyclic adenosine monophosphate
CAT: Catalase
Co Q10: Coenzyme Q10
CVD: Cardiovascular disease
DM: Diabetes mellitus
eNOS: Endothelial nitric oxide synthase
GLUT2: Glucose transporter 2
GPx: Glutathione peroxidase
HDL-C: High-density lipoprotein cholesterol
HO-1: Heme oxygenase 1
IDF: International Diabetes Federation
IRS: Insulin receptor substrate
Keap1: Kelch-like ECH-associated protein 1
LDL-C: Low-density lipoprotein cholesterol
MDA: Malondialdehyde
NADPH: Nicotinamide adenine dinucleotide phosphate
NO: Nitric oxide
NOX: NADPH oxidases
Nrf2: Nuclear factor erythroid 2-related factor 2
PPAR*α*: Peroxisome proliferator-activated receptor alpha
QUICKI: Quantitative insulin sensitivity check index
ROS: Reactive oxygen species
Sirt1: Sirtuin1
SOD: Superoxide dismutase
SREBPs: Sterol regulatory element-binding proteins
TAC: Total antioxidant capacity
TC: Total cholesterol
TG: Triglyceride
VLDL: Very low-density lipoprotein.
